# Post-cesarean section surgical site infection and associated factors in East Gojjam zone primary hospitals, Amhara region, North West Ethiopia, 2020

**DOI:** 10.1371/journal.pone.0261951

**Published:** 2021-12-31

**Authors:** Hulubante Bizuayew, Haimanot Abebe, Getachew Mullu, Likinaw Bewuket, Daniel Tsega, Tsegaw Alemye

**Affiliations:** 1 Department of Midwifery, Mizan Aman Health Science College, Mizan, Ethiopia; 2 Department of Nursing, Wolkite University, Wolkite, Ethiopia; 3 Department of Midwifery, Debre Markos University, Debre Markos, Ethiopia; 4 Department of Midwifery, Wolkite University, Wolkite, Ethiopia; University of Mississippi Medical Center, UNITED STATES

## Abstract

**Purpose:**

Maternal surgical site infection after cesarean delivery is a clinical problem which contributes to significant morbidity and mortality. In Ethiopia admissions following cesarean section due to surgical site infection have been routine activities of health care institutions but there is limited scientific evidence on both the magnitude of the problem and factors associated with it making prevention mechanisms less effective. Therefore, this study aimed to assess magnitude and risk factors of post-cesarean section surgical site infection at primary hospitals of East Gojjam Zone, Northwest Ethiopia.

**Methods:**

Institution-based cross sectional study with retrospective chart review was conducted from September 10–30 /2020 at 3 randomly selected primary hospitals of east Gojjam zone. The data were entered in Epi data version 3.1 and exported to Statistical Package for Social Science Software version 26. Post-cesarean section surgical site infection was measured based on disease classification and definition of the term by Center for Disease Control and Prevention. After checking for presence of multicollinarity, presence and degree of association of factors with outcome variable were computed through logistic regression analysis. Factors with P value ≤ 0.2 in bi-variable logistic regression analysis were included in the multivariable logistic regression analysis and those variables with P-value of <0.05 in multivariable analysis were considered statistically significant.

**Result:**

From 622 medical records of women who underwent cesarean section, 77 (12.4%) of them developed surgical site infection. Rural residence [(AOR = 2.30, 95%CI: (1.29, 4.09)], duration of labor greater than 24hrs [(AOR = 3.48, 95%CI: (1.49, 8.09)], rupture of membrane>12hrs[(AOR = 4.61,95%CI:(2.34,9.09)], hypertension[(AOR = 3.14,95%CI:(1.29,7.59)] and preoperative Hematocrit ≤30%[(AOR = 3.22,95%CI:(1.25,8.31)] were factors significantly associated with post-cesarean section surgical site infections.

**Conclusion:**

Magnitude of post-cesarean section surgical site infection was a significant problem in primary hospitals. Minimizing prolonged labor; minimize early rupture of membrane, properly managing patients with comorbidities like hypertension, strengthen prophylaxis and treatment for anemia during antenatal care and raising awareness for rural residents can reduce the problem. Zonal police makers should give emphasis to reduce its burden.

## Background

Cesarean section is an operative procedure by which a fetus, placenta, and membranes are delivered through an abdominal and uterine incision which is performed whenever abnormal conditions complicate labor and vaginal delivery that threatening the life or health of the mother or the baby [1]. In 1985, WHO declared that, the optimal threshold for cesarean section rate should be 10–15% [2]. But recent studies have reported that the rate of cesarean section is rising rapidly that leads to actual, potential, and life-long maternal and neonatal complications. Despite World Health Organization (WHO) recommended the optimal rate of cesarean section should lie between 5 and 15%, it is significantly increasing; even the reasons for the continued increase in the cesarean rates are not completely understood. Women having fewer children, maternal age is rising, use of electronic fetal monitoring is widespread, Malpresentation especially breech presentation, frequency of forceps and vacuum delivery is decreased, rate of labor induction increases, obesity dramatically raises and vaginal birth after cesarean section decreased are some of the possible explanations for increased incidence of cesarean section delivery. Despite cesarean section a lifesaving medical intervention and procedures to the decrease adverse birth outcome, controlling different postoperative neonatal and maternal complications are challenging in terms of patient safety; postpartum fever, surgical site infection, puerperal sepsis and maternal mortality are among common complications of CS [3].

One of the short term morbidity which takes place after CS is Surgical Site Infection (SSI). Globally, surgical site infections is potential complication associated with any type of surgical procedure, and is defined as infection which occurs within 30 days of a post-surgical procedure involving skin, subcutaneous tissue, soft tissue, or any other part of the body [4]. Mothers undergoing cesarean delivery have a 5 to 20-times greater chance of getting an infection compared with mothers who give birth vaginally [5]. Even though SSIs are among the most preventable hospital-acquired infections (HAI), it is a cause of morbidity and mortality among women undergoing cesarean section. Global reported rates of SSI were 3–15% with great burden on health-care systems, a person and communities at large, increasing the length of hospitalization, re-admission and costs of post-discharge care [6, 7]. High magnitude of surgical site infection following obstetric surgeries were reported in several African countries [8]. The magnitude of post cesarean section infection in the region ranges from 4.9% in Ruanda [9] to 12.5% in Nigeria [10]. In Ethiopia, the highest proportion of post CS SSI was reported in Addis Ababa and Mizan Tapi, which accounts for 15% and 12.9% respectively [11, 12]. Also, in Ethiopia SSIs were indicated as the commonest cause of hospital-acquired infection in Obstetrics and Gynecology ward than general surgical wards [13]. Since there is a great difference in health care quality and population served between specialized, general and primary hospitals, findings cannot be generalized. This study revealed the real prevalence and predictive factors of post-cesarean section surgical site infection at primary hospital level.

SSI is known to have both direct and indirect influences at individual, familial and health care system level [14]. For instance, the direct burdens of SSI includes but not restricted to postponed hospital stay, raised risk for readmission, prolonged antibiotic use, long term disability, and increased predisposition for depression and additional service charge including maternal death [15]. SSI also indirectly influences client’s functional and mental capacity and health-care service satisfaction that results in unproductivity. Patients who develop SSIs are more likely to spend their 60% of time in intensive care unit, readmitted to hospital and more likely to die compared to patients without surgical site infections [2].

According to previous studies factors that were associated with SSI after CS includes:- Being older age [16], previous CS [17], rural residency [18, 19], prolonged labor (24 or more hours) [19–21], premature rupture of membrane [19, 20, 22–24], gestational age<37weeks [23, 25], being multiparous [26–28], multiple vaginal examination [12, 24], longer duration of operation [29–31], longer post-operative hospital stay [11, 32], chronic anemia [33] and preexisting comorbidities like; diabetes mellitus, Hypertension and HIV AIDS [23, 24, 28, 30].

Ethiopian Federal Minister of Health highlighted several important measures to reduce SSI, called bundle of care. It includes appropriate use of antibiotics, appropriate pubic hair clipping, maintaining normal glucose levels in women with diabetics, and preventing hypothermia. Alternative measures, such as antisepsis, avoid unnecessary vaginal examination during labor, prepare skin with antiseptic agent immediately prior to surgery, administer prophylaxis antibiotic prior to incision and avoid manual removal of placenta, preoperative preparation, a reduction in the duration of surgery and reduction in blood loss [34]. Even though many efforts were tried by the government and non-government organizations in Ethiopia, a non-significant decline was achieved and post-cesarean section SSI is still a problem.

In Ethiopia admissions following CS due to SSI have been routine activities of health care institutions but there is limited scientific evidence on both the magnitude of the problem and factors associated with it making prevention mechanisms less effective. Even if there are some single centered studies in Ethiopia, the magnitude of SSI after CS greatly varies from hospital to hospital, even from ward to ward; this makes it difficult to generalize. In addition to this, studies conducted in our country were focused on single centered specialized and referral hospital [20] with no study conducted at primary hospital level in Ethiopia in general and East Gojjam zone in specific. This study also includes some variables that were not incorporated before, like number of doses of antibiotics, postoperative hematocrit count, and previous history of abortion. In general surgical site infection can serve as a measure of quality of hospital service [14] and this study aimed at determining the magnitude and risk factors that contribute for SSI following cesarean delivery at primary hospital level, which is a step ahead for preventing and reducing the problem. This study, together with other studies performed in the country, will inform policy makers about the magnitude of SSI among mothers who deliver by CS and enable them to prioritize and put national policies and procedures into action to combat the problem by considering factors associated with it.

## Material and methods

### Study design, setting and sampling

An institutional based cross sectional study based on one year retrospective chart review was conducted at 3 randomly selected primary hospitals of east Gojjam zone, Northwest Ethiopia from September10-30/2020 among mothers who underwent cesarean section from June 01, 2019 up to May 30, 2020. East Gojjam zone is one of the zones in Amhara regional state with the capital city of Debre Markos. The zone is bordered on the south by the Oromia region, on the West by West Gojjam, on the North by South Gondar, and on the East by South Wollo. East Gojjam Zone has 10 governmental Hospitals with one referral and nine primary hospitals with a catchment population of 3.8 million, based on Census conducted by central statically agency of Ethiopia (CSA) in 2007 [35]. The 3 selected hospitals was started cesarean section since 2016 but organized patient documentation was practiced science 2018 that can be traced for this study. The 3 hospitals provide surgical service with 1 general surgeon and 2 emergency surgeons for each. The minimum required sample size for the first objective was calculated by using single population proportion formula as n = (z α/2) * p (1-p)/ d^2^, by assuming prevalence (p) = 50% since there was no previous similar study at primary hospital level, 5% margin of error at 95% confidence interval and adding 10% for possible incomplete cards, finally multiplying by 1.5 for design effect it becomes 633.

The required sample size for second objective was calculated by considering various factors significantly associated with the outcome variable in previous studies assuming confidence interval level of 95%, Margin of error 5%, power 80%, and ratio of exposed to non-exposed as 1:1 using Epi-info software Stat Cal program and adding 10% for possible incomplete charts multiplied by 1.5 for design effect and taking the maximum value final sample size was taken as 633. A multistage systematic random sample technique was used to select the medical record of mothers who underwent CS. East Gojjam Zone has 9 primary hospitals, from those 3 hospitals namely Shegaw Mota primary hospital, Dejen primary hospital, and Bichena primary hospital were selected by lottery method. Number of medical records of women to be reviewed was proportionally allocated for each selected hospital, finally systematic random sampling technique using medical registration number as a sampling frame with calculated K value of two (k = 2) by selecting the first chart using lottery method was used to recruit medical records of mothers for each selected hospital.

### Operational definitions

Definition of surgical site infection in this study was based on the classification and definition of the term by Center for Disease Control and Prevention 1992 [36].

**Post-cesarean section surgical site infection**- is defined as the infection that occurred within the first 30 post-operative days and with at least one of the following signs and/ symptoms.

1. Purulent drainage from surgical site.
2. At least one of the following signs or symptoms of infection: pain or tenderness, localized swelling, redness, or heat
3. Surgical site abscess
4. Surgical site infection diagnosed by the surgeon or attending physician.

### Data collection tool and procedure

Data were extracted from the selected medical records of mothers undergoing CS using a pretested structured checklist developed after the review of similar literatures. The questionnaire has four parts: Socio-demographic variables, obstetric related factors, operation and anesthesia related factors and comorbidity variables. Data collection was made by 6 midwives and 3 supervisors after two day training session by the principal investigator. Two data collectors assigned for each hospital were traced and collected data from selected medical charts of women who undergo cesarean section from June, 2019 to May 30, 2020 using structured questionnaire and 1 supervisor for each hospital with the principal investigator supervise the whole data collection process. Surgical site infection was considered based on Center for Disease Control and Prevention (CDC) criteria. A post-CS SSI status was defined based on the Center for Disease Control and Prevention’s (CDC) standard. Post-cesarean section SSI was the outcome variable and was measured using four items with a yes/no response. The items used include: purulent discharge from surgical site, at least one sign of inflammation (pain/tenderness, localized swelling, redness or heat), abscess, and diagnosed wound infection by a physician/surgeon. A post-CS SSI was defined if there was at least one ‘yes’ response to any of the four items within 30 days after CS.

### Data quality control

To ensure quality of data, data collectors and supervisors were carefully trained about the objective of the study, the meaning of each alternative answers and blank spaces and when to skip as necessary. 5% pretest was done one week before the actual data collection period to ensure the validity of the questionnaire and modification was made accordingly. The collected data were reviewed and checked for completeness and consistency by the principal investigator daily. Proper coding and categorization of data was made before analysis.

### Data processing and analysis

The collected data were checked for completeness (i.e. incomplete charts were excluded), cleaned, coded and entered into Epidata version 3.1, and was exported to statistical package for social science software (SPSS) version 26, for analysis. The dependent variable was coded as 1 “for presence of post-cesarean section SSI”, 0 “if no SSI”. Descriptive statistics were computed and presented using text, frequency tables, graphs, percentage, mean and standard deviation followed by bi-variable and multivariable logistic regression analysis to determine statistical association between independent and outcome variables. Factors that had P-value of ≤ 0.2 in the bi-variable logistic regression analysis were fitted to multivariable logistic regression analysis. The strength of association of a particular variable was expressed by an adjusted odds ratio (AOR) with a 95% confidence interval. A two-tailed t-test P value of <0.05 was declared as a statistically significant. Moreover, variance inflation factor (VIF) and tolerance test was used to check for presence of multicollinarity and Hosmer and Lemeshow goodness of fit test was used to check for model fitness (0.86).

### Ethical consideration

Ethical approval was obtained from Mizan Aman Health Science College with ethical approval reference letter of HSc/R/C/Ser/Co/410/17/20. Permission was also obtained from East Gojjam zone health bureau and the respective heads of the maternity wards and record office head of the selected hospitals. All patient data were fully anonymized before data collection and confidentiality was assured throughout the study period and after a while. After informed, written and signed consent was obtained from medical directors and those responsible bodies’ to have data from patient medical records used in research, patient charts who underwent cesarean section from June 01, 2019 up to May 30, 2020 were selected and accessed from September 10–30 /2020, confidentiality of patient information was also ensured by data coding and omitting personal identifiers. All COVID-19 preventive measures were implemented throughout the data collection period.

## Results

### Socio-demographic characteristics

From a total of 633 selected medical records of mothers undergoing cesarean sections, 622 were eligible and reviewed in this study. The mean age of the mothers was 27.67 years with a standard deviation (SD) of 6 in a range of 16–45 years. The majority (64.3%) of them was less than 30 years old and more than half (58.2%) of the study participants were from rural settings **(Table 1).**

**Table 1:** Socio-demographic characteristics of women those who underwent cesarean section at primary hospitals of East Gojjam Zone, Northwest of Ethiopia, 2020.

Variables	Category	Frequency	Percent
Age	<30	400	64.3
	≥30	222	35.7
Religion	Orthodox	578	92.9
	Muslim	44	7.1
Residence	Urban	260	41.8
	Rural	362	58.2
Marital status	Married	609	97.9
	Others^a^	13	2.1
Educational status	Educated	305	49
	Uneducated	317	51

Key: others ^a^ (single, divorced, widowed).

### Obstetric related factors

Regarding parity, nearly half (49.2%) were multi-para and almost all the study participants (97.4%) had antenatal care follow-up. Women with prolonged premature rupture of membrane also accounted for only 15.6% of the participants and nearly two thirds (63.3%) had less than 24hr labor duration before operation. 1–4 times vaginal examination was performed for more than three fourths of women (81.7%) (**Table 2)**

**Table 2:** Obstetric related factors of mothers who underwent cesarean section at primary hospitals of East Gojjam zone, North Western Ethiopia, 2020.

Variables	Category	Frequency	Percent
Parity	Primi-Para	282	45.3
	Multi Para	306	49.2
	Grand multi Para	34	5.5
ANC visit	No	16	2.6
	Yes	606	97.4
Duration of labor	Not in labor	122	19.6
	<24hrs	394	63.3
	≥24hrs	106	17
Gestational age	<37wks	39	6.3
	37_40wks	490	78.8
	>40wks	93	15
Number of vaginal examinations	Not done	88	14.1
	1–4	508	81.7
	≥5	26	4.2
Duration of membrane rupture	Intact	285	45.8
	Ruptured<12hrs	240	38.6
	Ruptured≥12hrs	97	15.6
Present of meconium	Yes	132	39.1
	No	206	60.9
Present of chorioamninitis	Yes	24	7.1
	No	314	92.9
History of abortion	Yes	42	6.8
	No	580	93.2
History of previous C/S	Yes	68	10.9
	No	554	89.1

### Co-morbidity related factors

**Fig 1:**
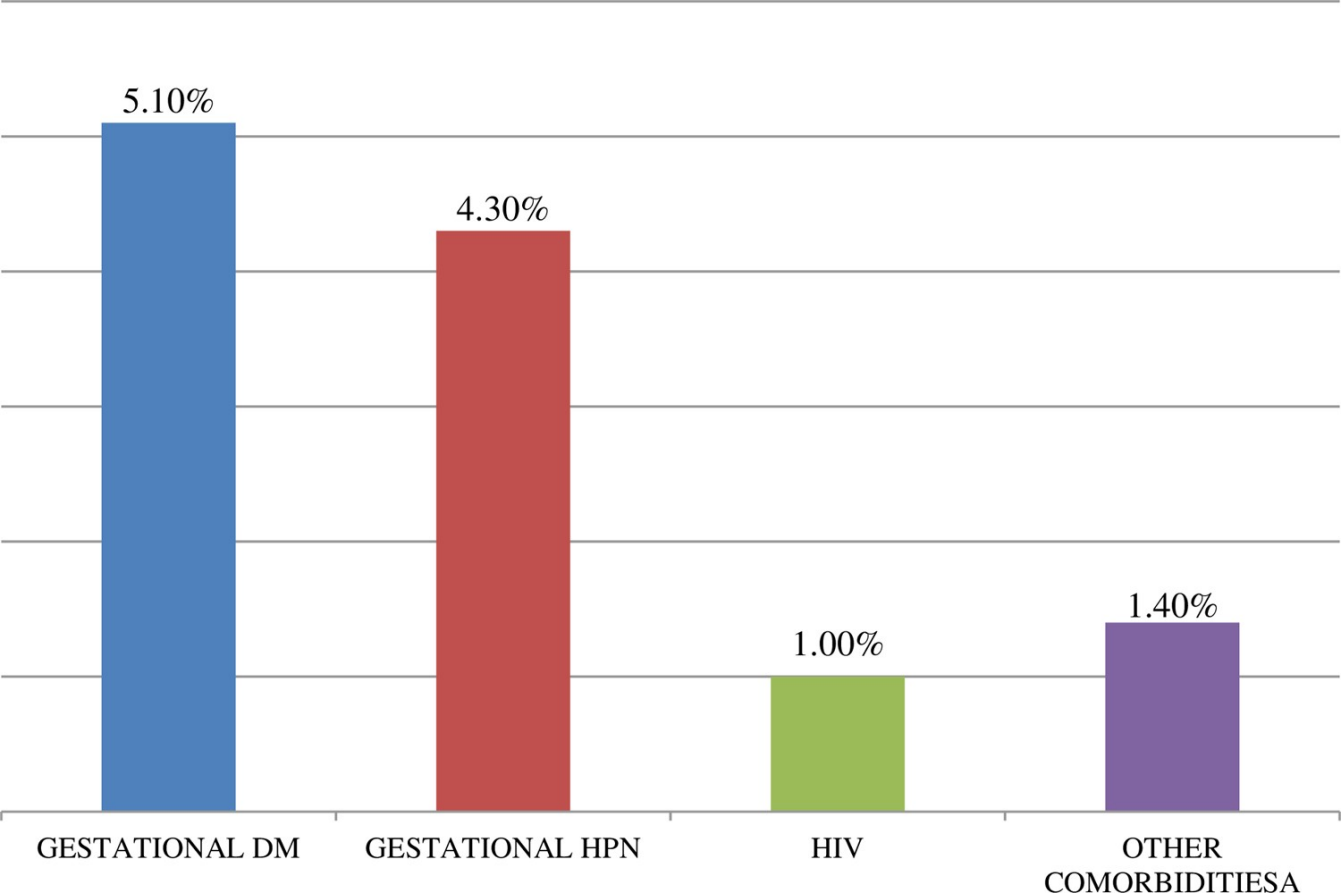
Co morbidity related factors of women who underwent cesarean section at primary hospitals of East Gojjam zone, North Western Ethiopia, 2020.

Among a total of 622 study participants, almost all (99%) of the study participants were non-reactive for HIV and 4.3% of them had gestational hypertension (**Fig 1)**.

### Operation and anesthesia related factors

Pre-operative antibiotics prophylaxis was provided for the great majority (95.8%) of the participants and post-operative antibiotics were given for all study participants as well as all women taking multiple dose(minimum 3-dose) of antibiotics after CS. Majority of participants (95.7%) had preoperative hematocrit of greater than 30% and above three fourths of them (80.1%) had less than 8 days of post-operative hospital stay **(Table 3)**. The highest indication for cesarean section was non-reassurance fetal heart rate (NRFHR) (27.33%) followed by cephalo-pelvic disproportion (CPD) (21.7%).

**Table 3:** Operation and anesthesia related factors of mothers who underwent cesarean section at primary hospitals of East Gojjam zone, North West Ethiopia, 2020.

Variables	Category	Frequency	Percent
Who perform the operation	Emergency surgeon	584	93.9
	Gynecologist	38	6.1
Preoperative HCT	≤30%	27	4.3
	>30%	595	95.7
Type of CS	Emergency	593	95.3
	Elective	29	4.7
Type of anesthesia	Regional	565	90.8
	General	57	9.2
Duration of operation	<60minutes	502	80.7
	≥60minutes	120	19.3
Type of abdominal incision	Transverse	608	97.7
	Vertical	14	2.3
Prophylactic antibiotics	Yes	596	95.8
	No	26	4.2
Post-operative antibiotics	Yes	622	100
Number of dose of antbiotics	Multiple dose	622	100
Post-operative HCT	≤30%	43	6.9
	>30%	579	93.1
Length of hospital stay	<8 days	498	80.1
	≥8 days	124	19.9
Blood transfusion	Yes	55	8.8
	No	567	91.2

### Magnitude of post cesarean section surgical site infection

**Fig 2:**
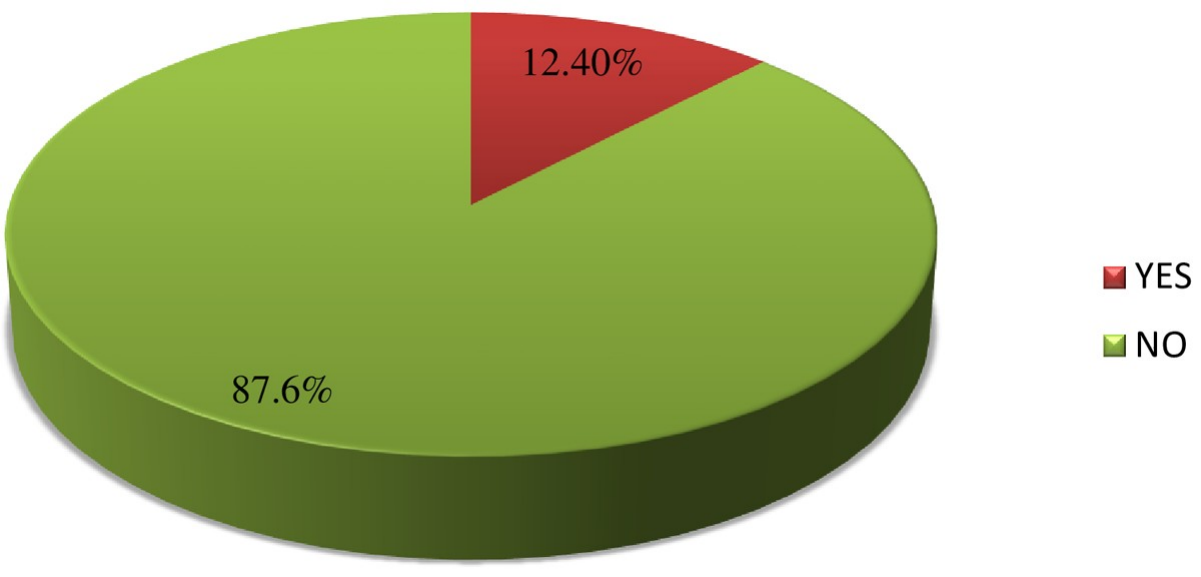
Magnitude of post cesarean section surgical site infection at primary hospitals of East Gojjam zone, North West Ethiopia, 2020.

Among 622 mothers included in the study, 77(12.4%) of them developed post-cesarean section surgical infection [95% CI: (9.8% - 14.9%)] **(Fig 2)**.

### Factors associated with post cesarean section surgical site infection

During bivariable logistic regression analysis, those variables which had P-value of ≤0.2 were considered in multivariable logistic regression. Out of thirty seven independent variables, ten variables, namely age, residence, parity, duration of rupture of membrane, duration of labor, length of hospital stay, hypertension, duration of operation, ante partum hemorrhage and preoperative HCT had shown an association in bi-variable analysis with p-value ≤ 0.2, and those variables were fitted into multivariable logistic regression analysis and in multivariable analysis rural setting, rupture of membrane greater than 12hrs, duration of labor greater than 24hrs, hypertension and preoperative HCT≤30% were significantly associated with post cesarean section surgical site infection at p–value < 0.05 with 95% confidence interval.

Women who were from rural areas were 2.3 times more likely to develop post-CS infection than those from an urban setting [AOR = 2.30, 95%C1: (1.295, 4.098; P = 0.005)]. The odds of developing post-CS infection among women who had a history of rupture of membrane greater than 12hrs before surgery were 4.6 times higher than those who had intact membrane[(AOR = 4.61, 95%CI:(2.336,9.094, P = 0.001)]. Women who had duration of labor greater than 24 hours were 3.5 times [(AOR = 3.48, 95%CI: (1.495, 8.086, P = 0.004)] more likely to develop post-CS infection than those who were not in labor. Similarly, hypertensive women were 3.1 times [(AOR = 3.14, 95%CI: (1.294, 7.593, P = 0.011)] more likely to have post-CS infection than non-hypertensive women. The odds of developing post CS infection among women who had preoperative HCT levels 30% and below were 3.2 times [(AOR = 3.22, 95%CI: (1.249, 7.8.309; P = 0.016)] as compared to the counterpart **(Table 4).**

**Table 4:** Bi-variable and multivariable logistic regression analysis of factors associated with post-cesarean section surgical site infection at primary hospitals of East Gojjam zone, northwestern Ethiopia, 2020.

Variables	Category	Post CS SSI		COR(95% CI)	AOR(95%CI	P-Value
		Yes	No			
Residence	Urban	20	240	1	1	
	Rural	57	305	2.243(1.311,3.836)**	2.304(1.295,4.098)	**0.005**
Membrane status	Intact	20	265	1	1	
	Ruptured<12hrs	28	212	1.75(.959,3.194)	1.427(0.752,2.706)	.277
	Ruptured≥ 12hrs	29	68	5.651(3.013,10.598)***	4.609(2.336,9.094)	**.001**
Labor status	Not labor	10	112	1	1	
	Labor ≤24 hrs.	36	358	1.126(.542,2.342)	.922(0.421,2.020)	.838
	Labor >24 hrs.	31	75	4.629(2.142,10.003)***	3.477(1.495,8.086)	**.004**
Parity	Primi-Para	28	254	1	1	
	Multi-Para	40	266	1.364(.817,2.278)	1.300 (0.695,2.433)	.411
	Grand-Para	9	25	3.266(1.387,7.687)**	2.2510(0.723,7.011)	.162
Duration of Operation	< 1hr	53	449	1	1	
	≥1hr	24	96	2.118(1.246,3.599)**	1.596(0.872,2.921)	.129
Age	<30 years	42	358	1	1	
	≥30 years	35	187	1.595(.985,2.584)	1.085(.574,2.050)	.803
Hypertension	Yes	9	23	3.067(1.363,6.901)**	3.135(1.294,7.593)	**.011**
	No	68	522	1	1	
APH	Yes	7	23	2.270(.939,5.483)	1.823 (.588,5.651)	.298
	No	70	522	1	1	
Preoperative HCT	≥30%	68	527	1	1	
	**<30%**	9	18	3.3.875(1.674,8.968)**	3.221(1.249,8.309)	**.016**
Length of hospital stay	<8 days	57	441	1	1	
	≥8 days	20	104	1.488(.856,2.585)	1.698(0.920,3.135)	.090

KEY:

** = P value < 0.01 and

*** = p value<0.001 at Bi variable analysis.

## Discussion

The aim of this study was to determine the magnitude and risk factors of post-cesarean section surgical site infection in East Gojjam zone primary hospitals. The study found that 12.4% [95% CI: (9.8%-14.9%)] of mothers who had CS developed surgical site infection. The figure might have been underestimated as the study was exclusively based on medical records review, and it did not involve post-discharge follow-up. Increased magnitude of post cesarean section surgical site infection in this study area can pose great health care burden in terms of economic/ financial loss to family and hospital due to prolonged hospitalization, increased workload for health care providers, risk of complication leading to maternal and neonatal mortality.

The finding of this study was in line with the global prevalence rate, which ranges from 3–15% [7]; a study in Nigeria (11%) and Tanzania (10.9%) [19, 30]. The reason might be due to similar quality in surgical procedures like routine use of prophylactic antibiotics that reduces risk for infection, all used retrospective chart review as means data collection, setting, in which those studies were conducted institutional based, comparable sample size as well as similarity in definition of SSI used to measure the outcome variable. This finding was also in line with other cross-sectional studies conducted in Ethiopia at Mizan tape (12.9%); Jimma (11.4%) and Hawassa Ethiopia (11%) [11, 18, 21] This might be due to similar means of data collection (retrospective chart review), post-cesarean section surgical site infection definition, and similar health care delivery system.

The finding was higher than other studies done in Pakistan (5.8%), Rwanda (4.9%), India (4.1%), Nepal (2.66%) [9, 32, 37, 38], and a study done in Israel-3.7% [16]. The possible reason for this discrepancy might be as a result of poor dietary habits and poor personal hygiene due to low socioeconomic status in our country, which predisposes mothers to nosocomial infection as a postoperative complication. In addition to this those studies were conducted at general and specialized hospitals with higher standard level of health care delivery and might be due to low standard of operating rooms in developing countries like Ethiopia than developed countries that leads to high risk of break in sterility technique during surgery. The finding of this study was still higher than a study done in Libya, which was 1.8% [17] which might be due to the difference in surgical site infection definition that used the presence of certain bacteria as a criteria to diagnose SSI which is more specific unlike the current study. Another possible justification for the discrepancy in magnitude of post-cesarean section surgical site infection as compared to some studies done in Ethiopia at Addis Ababa (8.4%), Debre Tabor (8%) and Bahir Dar (7.8%) [24, 39, 40] might be due to difference in population and study area. Those studies were conducted at referral and specialized hospitals located in more developed urban areas as compared to the current primary hospital level study serving many more rural residents with poor awareness, personal hygiene, low socioeconomic status and poor quality service due to the lack of specialized surgeons leading to increased magnitude of surgical site infections [20].

On the other hand, the magnitude of post-cesarean section surgical site infection in this study was less than a study done in Karachi tertiary care hospitals (24.3%) [41], Tanzania (48.2%) [42] and a prospective study conducted in Brazil (23.3%) [43]. This might be due to high prevalence of obesity and diabetes mellitus among Karachi women and lack of use of prophylactic antibiotics in this hospital which increases the risk of infection after surgery [41] and due to differences in study setting and design. Studies in Tanzania and Brazil were done at tertiary hospital level with increased referral cases having higher risk of infection as a postoperative complication and those studies were used prospective cohort study design with post discharge follow up in contrast to the current cross-sectional study with secondary chart review and no post-operative discharge follow-up that women may develop post cesarean section surgical site infection after discharge and may seek treatment to the nearby health facilities other than the study area that might underestimate the current magnitude.

In this study mothers from rural settings were 2.3 times more likely to develop post CS SSI than those women from urban areas, which was similar to study findings done in Saudi Arabia, Jimma and Tigray; Ethiopia [18, 28, 44]. The possible reason might be due to lack of awareness, poor dietary habits, poor personal hygiene, and low socioeconomic status in rural areas, which increases vulnerability to post-cesarean section surgical site infection [20].

Women who had duration of labor greater than 24hrs prior to CS were 3.5 times more likely to have surgical site infection than those who were not in labor, which was similar to findings done in Nigeria and Hawassa; Ethiopia [19, 21]. The possible explanation could be that as the duration of labor increases, the frequency of vaginal examinations also increases, which leads to ascending infections that might induce post-operative infection [12, 21, 24]. This implies that early detection of labor dystocia that might prolong labor must be detected early by midwives and surgeons necessary to reduce the time of exposure to infections prior to cesarean section.

Women who had premature rupture of membrane greater than 12hrs were 4.6 times more likely to develop post-cesarean section infection than those who were not. This finding was consistent with the study conducted in Nigeria and Addis Ababa; Ethiopia [19, 24]. Normally during pregnancy, cervical mucus plugs, fetal membranes and the amniotic fluid all serve as barriers to infection. However, when the membrane is ruptured, this protective effect is gradually reduced over time as amniotic fluid becomes no longer sterile. It is thought that the non-sterile amniotic fluid may act as a transport medium by which bacteria come into contact with the uterine and skin incisions leading to chorioamnionitis and may expose the mother to surgical site infection [45]. This implies that health education and guidance during the prenatal period should advocate preventing pregnant women from suffering from infections, such as vaginitis, in order to decrease morbidity from premature rupture of membranes.

Women with pregnancy-induced hypertension were about 3 times more likely to develop SSI than those mothers without the problem. This finding was consistent with the findings of previous studies done in Israel and Tanzania [23, 30]. The possible explanation might be hypo-perfusion of the wound caused by peripheral vasoconstriction effect of hypertension. In addition, those mothers with such problems might have edematous wound edges responsible for further entry of organisms and establishment of infection. Vascular disruption and high oxygen consumption by metabolically active cells may lead to oxygen depletion and hypoxic wound condition [40]. This implies that healthcare workers should monitor and manage hypertensive women before a cesarean section is performed.

In this study, preoperative HCT≤30% was significantly associated with post-cesarean section surgical site infection. Women with HCT≤30% were 3.2 times more likely to develop SSIs as compared to those with HCT>30%. This finding was in line with previous studies conducted in eight hospitals in Guangdong Province, China and Mizan tape; Ethiopia [11, 46]. Low HCT level reduces the oxygen tension at the wound site and increases the risk of SSI by compromising the activity of macrophages and delaying wound healing progress [47]. This implies that medical personnel should educate mothers during antenatal care and within the community about foods that are rich in iron and should also provide ferrous tablets to pregnant mothers.

## Conclusions

This study concludes that the magnitude of post-caesarean surgical site infection at primary hospitals of the east Gojjam zone was found to be higher, which was 12.40%. A list of factors including prolonged labor, prolonged rupture of membrane, rural setting, preoperative HCT count≤30% and gestational hypertension were significantly associated with post-cesarean section surgical site infection. Health care providers and zonal health policy makers should give emphasis on prevention of SSI to reduce its health care burden and associated mortality.

## Limitation of the study

Since it was a cross-sectional study, cause and effect relationship may not be established. In addition to that, the study was exclusively based on medical records review that lacks some variables, and it did not involve post-discharge follow-up so that the magnitude of the problem may be underestimated.

## Supporting information

S1 Data (SAV)

## List of abbreviations

ACOG: American College of Obstetrician and Gynecologists
AOR: Adjusted odd ratio
ANC: Antenatal care
CDC: Center for disease prevention and control
CS: Cesarean section
GA: Gestational age
HAI: Hospital acquired infection
HCT: Hematocrit
HIV: Human immune deficiency virus
HTN: Hypertension
NGO: Non- governmental organization
PROM: Premature ruptures of membrane
SPSS: Statistical Package for Social Science
SSA: Sub Saharan Africa
SSIs: Surgical site infections
WHO: World Health organization

## References

[1] Solomokin J, P G, P B, A L, S B, M E, et al. WHO global guidelines for the prevention of surgical site infection Geneva. Switzerland. Lancet Infect Dis. 2017;17(3):262–4. doi: 10.1016/S1473-3099(17)30081-6 28244389

[2] Betrán AP, Torloni MR, Zhang J-J, Gülmezoglu A, Section WWGoC, Aleem H, et al. WHO statement on caesarean section rates. BJOG: An International Journal of Obstetrics & Gynaecology. 2016;123(5):667–70. doi: 10.1111/1471-0528.13526 26681211 PMC5034743

[3] Betrán AP, Merialdi M, Lauer JA, Bing‐Shun W, Thomas J, Van Look P, et al. Rates of caesarean section: analysis of global, regional and national estimates. Paediatric and perinatal epidemiology. 2007;21(2):98–113. doi: 10.1111/j.1365-3016.2007.00786.x 17302638

[4] Alfouzan W, Al Fadhli M, Abdo N, Alali W, Dhar R. Surgical site infection following cesarean section in a general hospital in Kuwait: trends and risk factors. Epidemiology & Infection. 2019;147. 10.1017/S0950268819001675 PMC6805794 31597580

[5] Smaill FM, Grivell RM. Antibiotic prophylaxis versus no prophylaxis for preventing infection after cesarean section. Cochrane Database of Systematic Reviews. 2014(10). doi: 10.1002/14651858.CD007482.pub3 25350672 PMC8078551

[6] Allegranzi B, Bischoff P, de Jonge S, Kubilay NZ, Zayed B, Gomes SM, et al. New WHO recommendations on preoperative measures for surgical site infection prevention: an evidence-based global perspective. The Lancet Infectious Diseases. 2016;16(12):e276–e87. doi: 10.1016/S1473-3099(16)30398-X 27816413

[7] Saeed KB, Corcoran P, Greene RA. Incisional surgical site infection following cesarean section: A national retrospective cohort study. European Journal of Obstetrics & Gynecology and Reproductive Biology. 2019;240:256–60. doi: 10.1016/j.ejogrb.2019.07.020 31344664

[8] Grayi A, Vawdai Y. The development of national secondary legislation informing the implementation of the National Health Act continues apace. Health Policy and Legislation. 2018;21(23–46).

[9] Bizimana JK, Ndoli J, Bayingana C, Baluhe I, Gilson G, Habimana E. Prevalence and risk factors for post cesarean delivery surgical site infection in a teaching hospital setting in rural Rwanda: A prospective cross sectional study. International Journal of Current Microbiology and Applied Sciences 2016; 5(6):631–41.

[10] Onyegbule OA, Akujobi CN, Ezebialu IU, Nduka AC, Anahalu IC, Okolie V, et al. Determinants of post-caesarean wound infection in Nnewi, Nigeria. Journal of Advances in Medicine and Medical Research. 2015:767–74.

[11] Dacho A, Angelo A. Magnitude of post caesarean section surgical site infection and its associated factors among mothers who underwent caesarean section in Mizan Tepi University Teaching Hospital, South West Ethiopia, 2017. J Nurs Care. 2018;7(454):1168–2167.

[12] Hana Lijaemiro SBL, and Jembere Tesfaye Deressa. Incidence of Surgical Site Infection and Factors Associated among Cesarean Deliveries in Selected Government Hospitals in Addis Ababa, Ethiopia. Obstetrics and Gynecology International. 2020:, 8 pages. doi: 10.1155/2020/9714640 32148511 PMC7057000

[13] Daniel A, Zemanuel T. Hospital acquired surgical site and catheter related urinary tract infections among patients admitted in Mekele hospital, Mekele, Tigray, Ethiopia. AAU libraries electronic thesis and dissertation. 2008;23.

[14] Organization WH. Global guidelines for the prevention of surgical site infection: World Health Organization; 2016. 27929621

[15] Haque M, Sartelli M, McKimm J, Bakar MA. Health care-associated infections–an overview. Infection and drug resistance. 2018;11:2321. doi: 10.2147/IDR.S177247 30532565 PMC6245375

[16] Krieger Y, Walfisch A, Sheiner E. Surgical site infection following cesarean deliveries: trends and risk factors. The Journal of Maternal-Fetal & Neonatal Medicine. 2017;30(1):8–12. doi: 10.3109/14767058.2016.1163540 27023698

[17] Busarira M, Gahwagi M, Alagur N. Rate, indications and complications of caesarean section at Aljamahiriya Hospital, Benghazi, Libya. Journal of High Institute of Public Health. 2011;41(3):359–67.

[18] Amenu D, Belachew T, Araya F. Surgical site infection rate and risk factors among obstetric cases of Jimma University Specialized Hospital, Southwest Ethiopia. Ethiopian journal of health sciences. 2011;21(2):91–100. doi: 10.4314/ejhs.v21i2.69049 22434989 PMC3275863

[19] Ojiyi E, Dike E, Okeudo C, Ejikem E, Nzewuihe A. Wound Infection following Caesarean Section in a University Teaching Hospital in South-East Nigeria. Orient Journal of Medicine. 2013; 25(1–2):8–13.

[20] Adane F, Mulu A, Seyoum G, Gebrie A, Lake A. Prevalence and root causes of surgical site infection among women undergoing caesarean section in Ethiopia: a systematic review and meta-analysis. Patient safety in surgery. 2019;13(1):34. doi: 10.1186/s13037-019-0212-6 31673291 PMC6816205

[21] Wodajo S, Belayneh M, Gebremedhin S. Magnitude and factors associated with post-cesarean surgical site infection at Hawassa University teaching and referral hospital, southern Ethiopia: a cross-sectional study. Ethiopian journal of health sciences. 2017;27(3):283–90. doi: 10.4314/ejhs.v27i3.10 29217927 PMC5614999

[22] Chu K, Maine R, Trelles M. Cesarean section surgical site infections in sub-Saharan Africa: a multi-country study from Medecins Sans Frontieres. World journal of surgery. 2015;39(2):350–5. doi: 10.1007/s00268-014-2840-4 25358418 PMC4300431

[23] Schneid-Kofman N, Sheiner E, Levy A, Holcberg G. Risk factors for wound infection following cesarean deliveries. International Journal of Gynecology & Obstetrics. 2005;90(1):10–5. doi: 10.1016/j.ijgo.2005.03.020 15913620

[24] Worku M, Abdella A. Prevalence Of Surgical Site Infection And Associated Factors Among Mothers After Cesarean Delivery In Zewditu Memorial Hospital. Ethiopian Journal of Reproductive Health (EJRH. 2018;10(4).

[25] Mamo T, Abebe W.T and T. Y. Chichiabellu Risk factors for surgical site infections in obstetrics: a retrospective study in an Ethiopian referral hospital. patient safety in surgery. 2017(11):24. 28932266 10.1186/s13037-017-0138-9 PMC5605994

[26] Onsrud M, Sjøveian S, Mukwege D. Cesarean delivery-related fistulae in the Democratic Republic of Congo. International Journal of Gynecology and Obstetrics. 2011;114:10–4. doi: 10.1016/j.ijgo.2011.01.018 21529808

[27] Tsega F, Mengistie B, Dessie Y, Mengesha M. Prevalence of cesarean section in urban health facilities and associated factors in Eastern Ethiopia: hospital based cross sectional study. J Preg Child Health. 2015;2(3):169–73.

[28] Wendmagegn TA, Abera GB, Tsehaye WT, Gebresslasie KB, Tella BG. Magnitude and determinants of surgical site infecion among women underwent cesarean section in Ayder comprehensive specialized hospital Mekelle City, Tigray region, Northern Ethiopia, 2016. BMC pregnancy and childbirth. 2018;18(1):489. doi: 10.1186/s12884-018-2075-8 30541473 PMC6291995

[29] Jido T, Garba I. Surgical-site infection following cesarean section in Kano, Nigeria. Annals of medical and health sciences research. 2012;2(1):33–6. doi: 10.4103/2141-9248.96934 23209988 PMC3507120

[30] Mpogoro FJ, Mshana SE, Mirambo MM, Kidenya BR, Gumodoka B, Imirzalioglu C. Incidence and predictors of surgical site infections following caesarean sections at Bugando Medical Centre, Mwanza, Tanzania. Antimicrobial resistance and infection control. 2014;3(1):25. doi: 10.1186/2047-2994-3-25 25126415 PMC4131772

[31] Mulu W, Kibru G, Beyene G, Damtie H. Associated Risk factors for Postoperative Nosocomial infections among Patients admitted at Felege Hiwot Referral Hospital, Bahir Dar, Northwest Ethiopia. 2013;2(6):140–7.

[32] Vijayan C, Mohandas S, Nath AG. Surgical Site Infection Following Cesarean Section in a Teaching Hospital. International Journal of Scientific Study 2016.; 3(12):97–101.

[33] Harrison MS, Goldenberg RL. Cesarean section in sub-Saharan Africa. Harrison and Goldenberg Maternal Health, Neonatology, and Perinatology. 2016;2(6). 10.1186/s40748-016-0033-x PMC4937522 27398224

[34] Anderson DJ, Podgorny K, Berrios-Torres SI, Bratzler DW, Dellinger EP, Greene L, et al. Strategies to prevent surgical site infections in acute care hospitals: 2014 update. Infection Control & Hospital Epidemiology. 2014;35(S2):S66–S88. 25376070 10.1017/s0899823x00193869

[35] Macro O. Central Statistical Agency Addis Ababa, Ethiopia. 2006.

[36] Horan TC, Gaynes RP, Martone WJ, Jarvis WR, Emori TG. CDC definitions of nosocomial surgical site infections, 1992: a modification of CDC definitions of surgical wound infections. Infection Control & Hospital Epidemiology. 1992;13(10):606–8. 1334988

[37] Shrestha S, Wenju P, Shrestha R, Karmacharya R. Incidence and risk factors of surgical site infections in Kathmandu university hospital, Kavre, Nepal. Kathmandu University Medical Journal. 2016;14(54):107–11. 28166064

[38] Ansar A. Surgical site infection in obstetrics practice Pak J Surg. 2013;18: 2.

[39] Azeze GG, Bizuneh AD. Surgical site infection and its associated factors following cesarean section in Ethiopia: a cross-sectional study. BMC research notes. 2019;12(1):288. doi: 10.1186/s13104-019-4325-x 31133045 PMC6537424

[40] Molla M, Temesgen K, Seyoum T, Melkamu M. Surgical site infection and associated factors among women underwent cesarean delivery in Debretabor General Hospital, Northwest Ethiopia: hospital based cross sectional study. BMC Pregnancy and Childbirth. 2019; 19(1):317. doi: 10.1186/s12884-019-2442-0 31464598 PMC6716814

[41] Jabbar S, Perveen S, Naseer Q. Surgical site infection (SSI): frequency and risk factors in post caesarean section cases in a tertiary care hospital. ASH KMDC. 2016;21(4):233–9.

[42] De Nardo P, Gentilotti E, Nguhuni B, Vairo F, Chaula Z, Nicastri E, et al. Post-caesarean section surgical site infections at a Tanzanian tertiary hospital: a prospective observational study. Journal of Hospital Infection. 2016;93(4):355–9. 10.1016/j.jhin.2016.02.021 27125664

[43] Del Monte MCC, Neto AMP. Postdischarge surveillance following cesarean section: the incidence of surgical site infection and associated factors. American journal of infection control. 2010;38(6):467–72. doi: 10.1016/j.ajic.2009.10.008 20226571

[44] Tran TS, Jamulitrat S, Chongsuvivatwong V, Geater A. Risk Factors for Post cesarean Surgical Site Infection. Obstetrics & Gynecology. 2000;95(3):367–71. 10711546 10.1016/s0029-7844(99)00540-2

[45] Wager GP, Martin DH, Koutsky L, Eschenbach DA, Daling JR, Chiang W, et al. Puerperal infectious morbidity: relationship to route of delivery and to antepartum Chlamydia trachomatis infection. American journal of obstetrics and gynecology. 1980;138(7):1028–33. doi: 10.1016/0002-9378(80)91102-3 7468665

[46] Gong SP, Guo HX, Zhou HZ, Chen L, Yu YH. Morbidity and risk factors for surgical site infection following cesarean section in Guangdong Province, China. Journal of Obstetrics and Gynaecology Research. 2012;38(3):509–15. doi: 10.1111/j.1447-0756.2011.01746.x 22353388

[47] Gordillo GM, Sen CK. Revisiting the essential role of oxygen in wound healing. The American journal of surgery. 2003;186(3):259–63. doi: 10.1016/s0002-9610(03)00211-3 12946829

